# Ricord and His Patient

**Published:** 1856-07

**Authors:** 


*Ricord and his Patient.*—The following sprightly feuilleton is from the pen of Dr. Schlesinger, a Paris correspondent of the *Vienna Medical Times:*

"I had an opportunity of observing a case in the private practice of Prof. Ricord, and the communication may not be uninteresting, when viewed from the standpoint of *unus pro multis.* A young countryman had enjoyed the seductive charms of beautiful Lutaetia, and had studied the manner of living of the French, particularly in those large schools at Mabile, Jardin, d'Hiver, Asnières, and indeed had become so immersed in his favorite study, that he bore away, as a final prize—a gonorrhœa. The martyr to these studies had already been, when I arrived in Paris, for seven weeks in undisputed possession of this enviable acquisition. Ricord was his physician. During this time he had made twenty visits to Ricord, and received six visits from him. For the former, Ricord received each time the usual honorarium of twenty francs — the latter forty francs. The Doctor's bill alone was 640 francs.— But the gonorrhœa, at the end of the seventh week, was still—*in floribus.* Besides the Doctor, the apothecary had profited, in this right French article, from 120 to 150 francs. Ricord writes recipes, many, very many recipes, daily changing them, and daily making them more costly. He says, you can have them put up by any favorite apothecary, "*mais le Pharmacien Favrot, Rue Richelieu, connoit, déja mes ordonnances.*" The hint is as good as a command. You go then to Favrot, Rue Richelieu. And our countryman went there also. Ricord had prescribed already, by turns, tisans, *limonade gazeuse purgative, capsules de cubèbe, capsules de copahu, copahine mége ferrée prep.*, different syrups, baths, and costly perfumed injections, when, in addition to the gonorrhœa, slight symptoms of the commencement of an epididymitis appeared, and he ordered the application of fifteen leeches. The Pharmacien Favrot, Rue Richelieu, is a French tradesman, in the most gallant and refined sense of this significant word. He takes the recipe, and tells the young man that he will send the leeches, with suitable accompaniments, to the hotel. Now comes a pretty demoiselle with an elegant *carton.* Therein are to be fo nd leeches, linen, charpie, sponge, sticking plaster, suspensory, syringe, scissors—in short, everything that a man in such a condition could desire for weal or woe, and everything is nice and tasteful. The pretty bearer of this Pandora's box is the "*Nourrice.*" The Nourrice is the attendant, the leecher. The young man is taken aback,—the feeling of shame suddenly comes to him. She discovers it and says: "*Eh! vous avez honte allez vous en' j'ai dejà vu ça depuis mon enfance mille et mille fois.*" I have cofidence in her veracity.

In addition to the usual quick irritability of the genitals during a gonorrhœa, the pretty nourrice, although she understands how to treat the *corpus delecti,* with practiced and very delicate hands, yet still remains a — pretty nourrice. The nourrice laughs maliciously, and relates, during the application, some most charming little stories out of the *Chronique Scandaleuse* of the Parisians. She knows many a piquant tale even about the Doctor and the apothecary.

After a few days, the apothecary's bill comes in, 80 to 100 francs for the carton, its contents, and the messenger. I have in my possession such recipes and bills. The circumstance that the apothecaries in France, as in England, have a care for everything that a man requires in such a gallant condition, has also its bright side, since the patient is not obliged to run to ten places before he collects his curative apparatus. This makes a wearisome day. In our country, the apothecary would be sued for damage to business, on account of the linen by the linen draper, of the sponge by the grocer, of the knife by the cutler, of the suspensory by the bandage-maker, of the syringe by the glass-blower or pewter-worker, and the Board of Trade and the magistrate would have their trouble!

After I had been in Paris five weeks, in the twelfth of the gonorrhœa, the young man was still not rid of his dear companion —he was very morose, not able to continue his studies, and had expended there a sum of 1,200 francs on the doctor and the apothecary. A pretty outlay of capital, with—*running* interest."—*Am. Med. Monthly.*

